# Cdc42-Interacting Protein 4 Represses E-Cadherin Expression by Promoting β-Catenin Translocation to the Nucleus in Murine Renal Tubular Epithelial Cells

**DOI:** 10.3390/ijms160819170

**Published:** 2015-08-14

**Authors:** Chuou Xu, Qiaodan Zhou, Lili Liu, Ping Liu, Guangchang Pei, Rui Zeng, Min Han, Gang Xu

**Affiliations:** Division of Nephrology, Department of Internal Medicine, Tongji Hospital, Tongji Medical College, Huazhong University of Science and Technology, 1095 Jiefang Ave., Wuhan 430030, China; E-Mails: xchuou@163.com (C.X.); qiaodanzhou_1000@163.com (Q.Z.); lilyliu1637@sina.com (L.L.); liup1986@tom.com (P.L.); pgc2008@sina.com (G.P.); zengr126@126.com (R.Z.); minhan@tjh.tjmu.edu.cn (M.H.)

**Keywords:** TGF-β1, CIP4, β-catenin, E-cadherin, renal tubular epithelial cells

## Abstract

Renal fibrosis is an inevitable outcome of end-stage chronic kidney disease. During this process, epithelial cells lose E-cadherin expression. β-Catenin may act as a mediator by accumulation and translocation to the nucleus. Studies have suggested that CIP4, a Cdc42 effector protein, is associated with β-catenin. However, whether CIP4 contributes to E-cadherin loss in epithelial cells by regulating β-catenin translocation is unclear. In this study, we investigated the involvement of CIP4 in β-catenin translocation. Expression of CIP4 was upregulated in renal tissues of 5/6 nephrectomized rats and mainly distributed in renal tubular epithelia. In TGF-β1-treated NRK-52E cells, upregulation of CIP4 expression was accompanied by reduced expression of E-cadherin. CIP4 overexpression promoted the translocation of β-catenin to the nucleus, which was accompanied by reduced expression of E-cadherin even without TGF-β1 stimulation. In contrast, CIP4 depletion by using siRNA inhibited the translocation of β-catenin to the nucleus and reversed the decrease in expression of E-cadherin. The interaction between CIP4 and β-catenin was detected. We also show that β-catenin depletion could restore the expression of E-cadherin that was suppressed by CIP4 overexpression. In conclusion, these results suggest that CIP4 overexpression represses E-cadherin expression by promoting β-catenin translocation to the nucleus.

## 1. Introduction

Cdc42 (cell division cycle 42 (GTP binding protein)) is a member of the Rho family of small GTPases and is an important regulator of actin polymerization and cytoskeleton reorganization. Cdc42-interacting protein 4 (CIP4), a downstream effector of Cdc42, is a 545-amino acid protein that can bind to Cdc42 and Wiskott-Aldrich syndrome protein (WASP) through a Cdc42-binding domain and an SH3 domain at the carboxyl terminus, and also contains a FER/CIP4 homology (FCH) domain, through which it interacts with tubulin [1,2,3]. Rho-like GTPases are also key players in transforming growth factor-β1 (TGF-β1)-induced non-Smad signaling pathways [4,5].

Recent studies showed that TGF-β1 may induce loss of E-cadherin expression in certain epithelial cells [6]. E-cadherin normally operates as a homophilic adhesion receptor to facilitate cell–cell recognition and adhesion [7]. The ectodomains of E-cadherin on adjacent cells bind to each other [8,9], while the cytoplasmic domains interact with β-catenin (a kind of arm-repeated protein). β-Catenin directly binds to E-cadherin and is crucial to its adhesive function [7,10]. However, under pathological conditions such as tissue fibrosis or cancer, the interaction between cytoplasmic domains of E-cadherin and β-catenin are destroyed, leading to impairment of cell–cell adhesion [11]. Furthermore, free β-catenin translocates to the nucleus and functions as a mediator by binding to target genes such as *Slug*, a member of the *Snail* family, repressing the expression of E-cadherin [12,13].

There is evidence that CIP4 has a physical connection with β-catenin and is critical for cell–cell adhesion in renal cell carcinoma [2]. However, whether CIP4 contributes to E-cadherin loss induced by TGF-β1 or has an effect on E-cadherin expression is still unknown. To assess this potential contribution, we measured CIP4 expression in both renal tissues of 5/6 nephrectomized rats and renal tubular epithelial cells after TGF-β1 treatment, and examined the effect of CIP4 on β-catenin translocation to the nucleus and E-cadherin expression* in vitro*.

## 2. Results

### 2.1. Distribution and Expression of CIP4 in Renal Tissue of 5/6 Nephrectomized Rats

**Figure 1:**
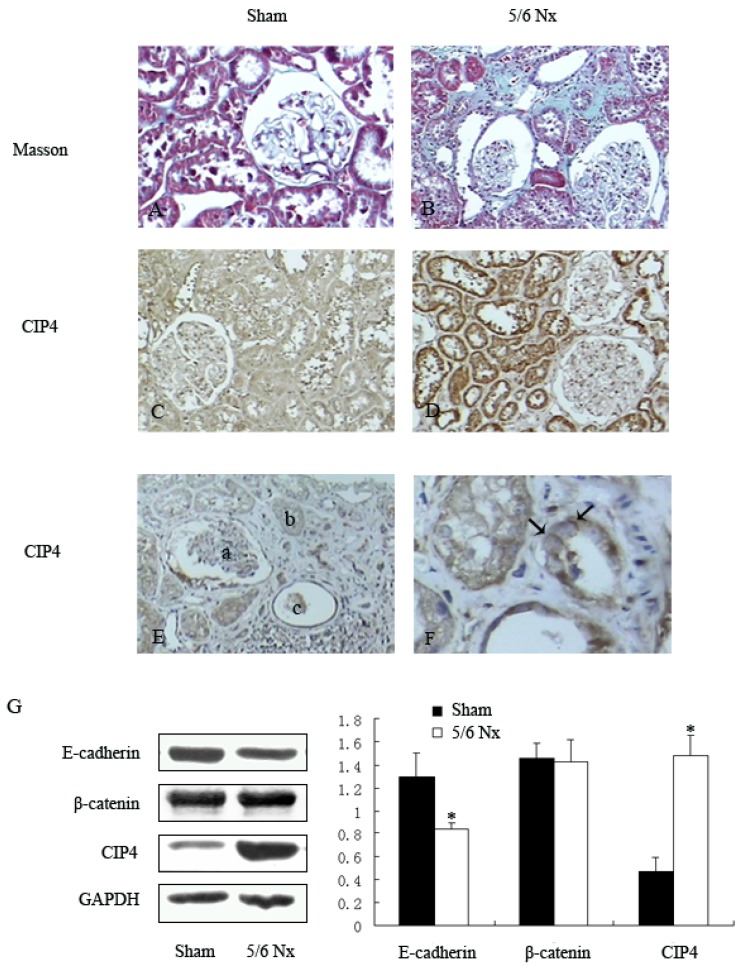
Expression and distribution of CIP4 in fibrotic renal tissue of rats: (**A**,**B**) analysis of tissue fibrosis by staining of kidney sections with Masson’s trichrome stain, 200×; (**C**,**D**) immunohistochemical analysis of CIP4 expression in the renal tissue of 5/6 nephrectomized and sham-operated rats, 200×; (**E**) representative staining of CIP4 distribution in the area of the glomerulus (a), renal tubule (b) and blood vessel (c), 200×; (**F**) CIP4 is mainly distributed at the basolateral side of epithelial cells (black arrows), 400×; and (**G**) Western blot of E-cadherin, β-catenin, and CIP4 in renal tissues of sham-operated and 5/6 nephrectomized rats. The histogram shows the average volume density normalized to the loading control GAPDH, (*n* = 3); * *p* < 0.05 compared with sham-operated rats.

We first examined the distribution of CIP4 in fibrotic renal tissue in the 5/6 subtotal nephrectomy model. Masson staining revealed glomerular sclerosis and interstitial fibrosis in the renal tissues of the 5/6-nephrectomized rats, but not in the renal tissues of the sham-operated rats (Figure 1A,B). Immunohistochemical staining showed that CIP4 was highly expressed in renal tubular epithelia of 5/6-nephrectomized rats (Figure 1C,D), expressed in much lesser amounts in blood vessels or glomeruli, and rarely observed in tissues from sham-operated rats (Figure 1E). It was interesting to find that CIP4 was mainly distributed at the basolateral side of renal tubular epithelia (Figure 1F).

Decreased E-cadherin expression has been found in kidney specimens from patients who suffered from glomerulonephritis or diabetic or other forms of chronic nephropathy. It was also reported that the E-cadherin and β-catenin complex was responsible for adherens junctions [7]. Therefore, we examined the expressions of E-cadherin, β-catenin, and CIP4 in renal tissues of sham-operated and 5/6 nephrectomized rats. Compared to the sham-operated group, the expression of CIP4 was increased (3.38-fold) in the renal tissue of 5/6 nephrectomized rats, and this was accompanied by reduced expression of E-cadherin (*p* < 0.05), while there were no differences between the two groups in β-catenin expression (Figure 1G).

### 2.2. TGF-β1 Increases Expression of CIP4 and Induces CIP4 and β-Catenin Translocation to the Nucleus in the NRK-52E Cell Line

**Figure 2:**
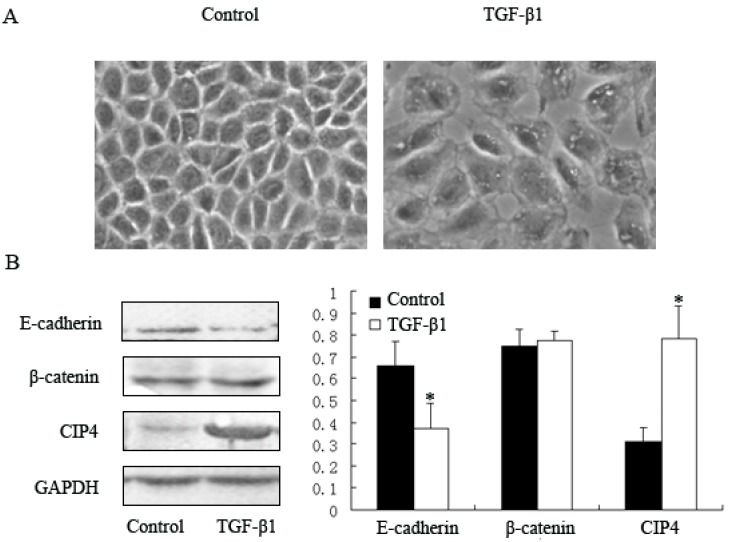

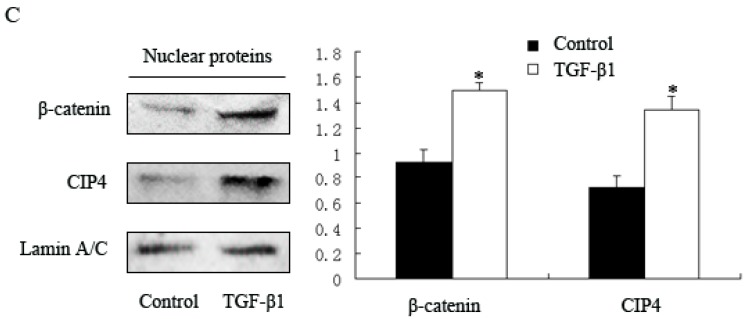
CIP4 is upregulated in TGF-β1**-**treated NRK-52E cells. (**A**) Phase contrast microscopy showing morphological changes in TGF-β1 treated cells, 100×; Western blots for protein expression of (**B**) E-cadherin, β-catenin and CIP4 in whole cell lysates; and (**C**) β-catenin and CIP4 in nuclear proteins of control and TGF-β1-treated cells. Histograms show the average volume density normalized to the loading controls GAPDH (**B**) and Lamin A/C (**C**), (*n* = 3); * *p* < 0.05 compared with control cells.

Given that CIP4 was mainly distributed in renal tubular epithelia, we examined the expression of CIP4 in a renal epithelial cell line derived from rat proximal tubular cells (NRK-52E cells). TGF-β1 has been reported to cause reduced expression of E-cadherin in various types of epithelial cells, which is relevant to the pathogenesis of renal fibrosis [6,11,14]. We further investigated potential changes in CIP4 expression in TGF-β1-treated NRK-52E cells. Compared with cells from the control group, TGF-β1-treated NRK-52E cells were larger after a 72-h incubation with 10 ng/mL TGF-β1 and had loose connections between cells (Figure 2A). Western blot showed that CIP4 was significantly elevated (2.53-fold) in the TGF-β1-treated group; this was accompanied by a decrease in expression of E-cadherin (*p* < 0.05). There were no differences between these two groups in β-catenin expression (Figure 2B).

We detected increased expression of CIP4 in both renal tissue of 5/6 nephrectomized rats* in vivo* and TGF-β1 treated NRK-52E cells* in vitro*. This was accompanied by decreased expression of E-cadherin, but not of β-catenin. Some studies have suggested that nuclear translocation of β-catenin could be observed during fibrosis in addition to the loss of E-cadherin expression [7,14]. We next investigated the expression of CIP4 and β-catenin in nuclear fractions. Nuclear expression of CIP4 and β-catenin in TGF-β1-treated cells was 1.88- and 1.63-fold greater, respectively, than in control cells (Figure 2C).

### 2.3. CIP4 Interacts with β-Catenin and Overexpression of CIP4 Induces E-Cadherin Downregulation in NRK-52E Cells

**Figure 3:**
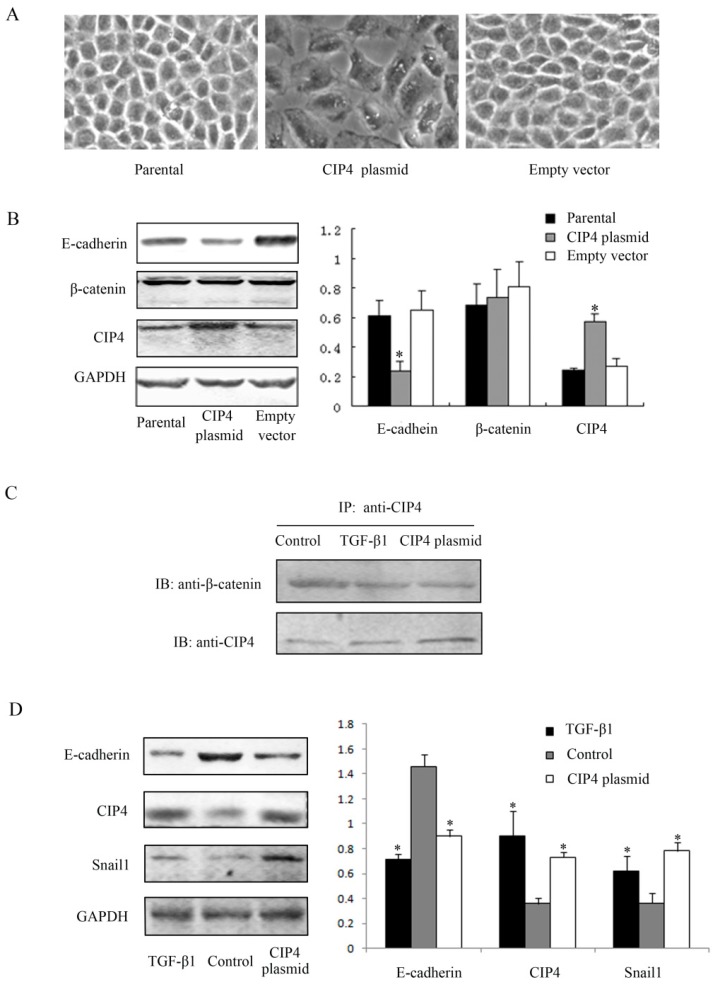
Overexpression of CIP4 in NRK-52E cells is accompanied by decreased expression of E-cadherin. (**A**) Morphological changes of CIP4 plasmid-transfected cells compared with parental and empty vector-transfected cells, 100×; (**B**) Western blots for protein expression of E-cadherin, β-catenin and CIP4 in parental, CIP4 plasmid-transfected and empty vector-transfected cells. The histogram shows the average volume density normalized to the loading control GAPDH (*n* = 3); * *p* < 0.05 compared with parental cells; (**C**) Immunoprecipitation for interaction of β-catenin and CIP4; and (**D**) Western blots for protein expression of E-cadherin, CIP4 and Snail1 in TGF-β1 treated cells, control cells and CIP4 plasmid-transfected cells. The histogram shows the average volume density normalized to the loading control GAPDH (*n* = 3); * *p* < 0.05 compared with control cells.

Rho-like GTPases participate in a critical pathway of TGF-β1 signaling [4,5] and are involved in the regulation of adherens junction function in epithelial cells. Moreover, CIP4 has been reported to promote migration and invasion of lung cancer cells [9,15]. Thus, we examined whether CIP4 contributes to E-cadherin loss of epithelial cells. Compared with parental cells and cells transfected with empty vectors, protein expression of CIP4 was 2.38- and 2.07-fold greater, respectively, in cells transfected with CIP4 plasmids (Figure 3B). Light microscopy showed that compared to control cells, cells transfected with CIP4 plasmids were larger and more disassociated from surrounding cells, just as we observed in TGF-β1 treated cells (Figure 3A). The epithelial marker E-cadherin was downregulated in cells that overexpressed CIP4, compared with parental cells and empty vector-transfected cells (*p* < 0.05). We then investigated protein expression of β-catenin, a component of the intercellular adhesive junction [8]. There were no differences among the three groups of cells. As mentioned above, CIP4 was mainly distributed on the basolateral side of renal tubular epithelia, which is also where β-catenin is located [16]. Therefore, we further examined the interaction between CIP4 and β-catenin. Our results showed they interacted with each other not only in parental cells, but also in TGF-β1-treated and CIP4-transfected cells (Figure 3C). Furthermore, in those three groups of cells mentioned above, increased expression of Snail1 was detected by western blot in TGF-β1 treated cells and CIP4-transfected cells accompanied by reduced expression of E-cadherin and increased expression of CIP4 (*p* < 0.05) (Figure 3D).

### 2.4. CIP4 Depletion Reverses the Decreased Expression of E-Cadherin and Regulates Translocation of β-Catenin to the Nucleus of NRK-52E Cells

**Figure 4:**
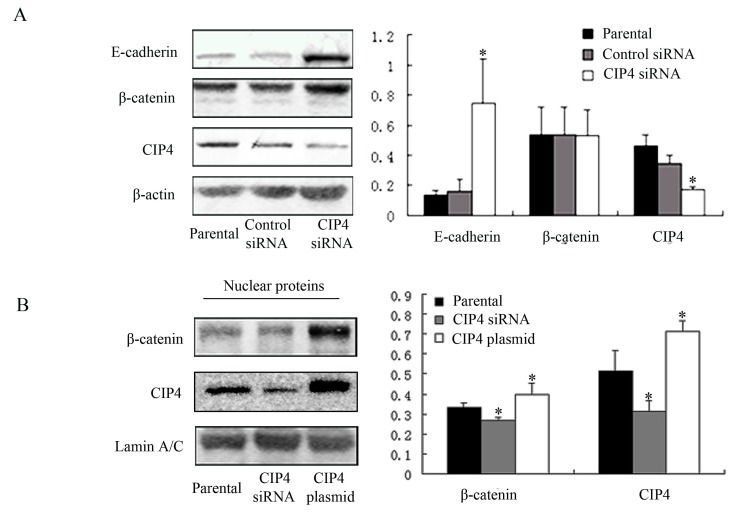
CIP4 depletion reverses the expression of E-cadherin and regulates translocation of β-catenin. Western blots for protein expression of (**A**) E-cadherin, β-catenin, and CIP4 in whole cell lysates of parental, control siRNA-transfected, and CIP4 siRNA-transfected cells; and (**B**) β-catenin and CIP4 in nuclear proteins of parental, CIP4 siRNA-transfected, and CIP4 plasmid-transfected cells. Histograms show the average volume density normalized to the loading control β-actin in (**A**) and Lamin A/C in (**B**), (*n* = 3); * *p* < 0.05 compared with parental cells.

Due to the results above, we wondered whether CIP4 depletion could affect E-cadherin expression. We examined E-cadherin expression after CIP4 depletion by transfection with CIP4-siRNA. We found that CIP4 expression was only 50% of the control after CIP4 depletion (Figure 4A). E-cadherin expression in CIP4-depleted cells was dramatically increased compared with control cells (*p* < 0.05).

Nuclear translocation and accumulation of β-catenin are known to activate specific target genes [12,13]. In our study, we found that β-catenin was upregulated in nuclear fractions of cells overexpressing CIP4, while it was downregulated in CIP4-depleted cells (*p* < 0.05; Figure 4B). These results demonstrate that CIP4 can regulate nuclear translocation of β-catenin.

### 2.5. β-Catenin Depletion in Cells Overexpressing CIP4 Restores the Expression of E-Cadherin

**Figure 5:**
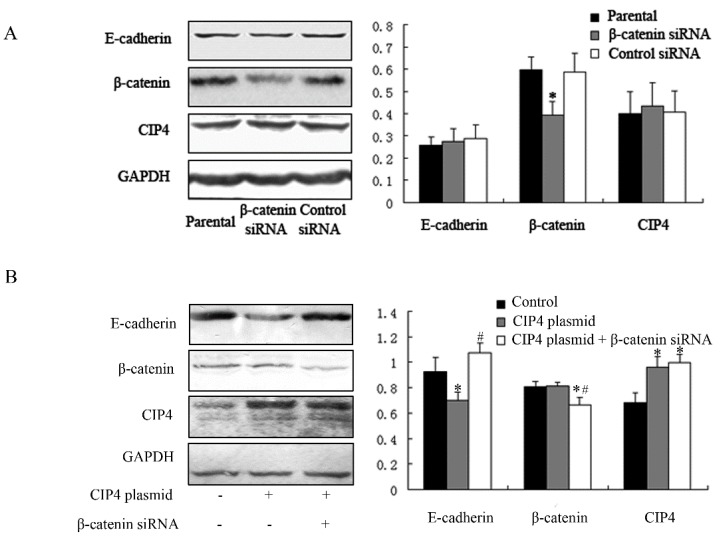
CIP4 regulates E-cadherin expression through β-catenin. (**A**) Western blots for protein expression of E-cadherin, β-catenin, and CIP4 in parental, β-catenin siRNA-transfected, and control siRNA-transfected cells; Cells overexpressing CIP4 were transfected with β-catenin siRNA for β-catenin depletion and cell lysates were analyzed by (**B**) Western blot for protein expression of E-cadherin, β-catenin and CIP4. The histograms show the average volume density normalized to the loading control GAPDH (*n* = 3); * *p* < 0.05 compared with parental cells; # *p* < 0.05 compared with CIP4 plasmid-transfected cells.

β-Catenin is known to be part of the adhesive complex of epithelial cells [7,8] and associates physically with CIP4 [2]. Thus, we next investigated whether β-catenin depletion had an effect on E-cadherin expression. β-Catenin expression was reduced by β-catenin-specific siRNA (*p* < 0.05), and neither the expression of E-cadherin nor that of CIP4 was different from that of parental cells or control siRNA-transfected cells (Figure 5A). Furthermore, β-catenin depletion restored the expression of E-cadherin in cells overexpressing CIP4 (Figure 5B). Thus, we conclude that CIP4 overexpression represses E-cadherin expression by promoting β-catenin translocation to the nucleus.

## 3. Discussion

TGF-β1 is a pleiotropic cytokine that is critical for various processes as well as for various physiological processes and pathological conditions [5,11]. To regulate these functions, TGF-β1 uses multiple intracellular signaling pathways such as the canonical TGF-β/Smad, and cross-talk with non-Smad signaling pathways. The non-Smad pathways include various branches of MAP kinase (MAPK) pathways, Rho-like small GTPase signaling pathways, and phosphatidylinositol-3-kinase (PI3K)/Akt pathways [4,5,17,18]. TGF-β1 is also known as an inducer of the epithelial-mesenchymal transition (EMT), in which epithelial cells lose E-cadherin expression [14]. There is some debate as to the contribution of EMT to tissue fibrogenesis [19]. In the present study, we found decreased E-cadherin expression in fibrotic renal tissues of 5/6 nephrectomized rats, confirming the loss of E-cadherin in the* in vivo* model. CIP4 expression was also upregulated and mainly distributed at the basolateral side of renal tubular epithelia, where E-cadherin/β-catenin is located [16]. We did not know whether these results were due to the process of EMT, but CIP4 appeared to have an effect on E-cadherin expression. Additional measurements of EMT markers, such as alpha-smooth muscle actin (α-SMA) or fibroblast-specific protein 1 (FSP), could clarify the potential role of EMT in the present model. *In vitro*, we also found that NRK-52E cells exhibited morphological changes together with E-cadherin loss after TGF-β1 stimulation, similar to what occurs in the process of EMT.

Recent investigations show that Rho-like small GTPases may initiate the TGF-β1 signaling pathways by regulating receptor endocytosis, Smad trafficking, and actin cytoskeleton remodeling [4,5]. Cdc42 belongs to the Rho family of small GTPases and is involved in the regulation of E-cadherin activities [1,9]. CIP4, which is a downstream target protein of Cdc42, acts as a link between Cdc42 signaling and the regulation of cytoskeletal proteins [1,3]. Cytoskeletal rearrangement is required for the migration and invasion of malignant cells, such as lung cancer cells. CIP4 overexpression may lead to cytoskeletal reorganization and contribute to the metastatic phenotype [15]. In our study, CIP4 was upregulated in TGF-β1-treated NRK-52E cells, just as we observed in 5/6 nephrectomized rats.

E-cadherin mediates cell–cell interactions and plays key roles in the establishment and maintenance of tissue architecture. Loss of E-cadherin is consistently observed in tumor malignancy and in many kinds of chronic kidney diseases, such as autosomal dominant polycystic kidney disease (ADPKD) and diabetic nephropathy, resulting in tissue fibrosis [7,20,21]. We also found E-cadherin loss in the renal tissues of 5/6 nephrectomized rats and TGF-β1-treated NRK-52E cells. E-cadherin, a member of the classic cadherin family, together with β-catenin and α-catenin, form the core adhesive complex in epithelia, which is important for their adhesive function [7,8]. β-Catenin is an excellent candidate to mediate signals regulating cadherin adhesive contacts. For example, β-catenin can directly bind to several signaling proteins [12,13], tyrosine phosphorylation of β-catenin is associated with changes in intercellular adhesion [22], and β-catenin can act as a transcriptional coactivator, enhancing the transcription of genes such as those in the* Snail* gene family and thereby regulating expression of E-cadherin [12,23,24]. Tsuji* et al.* [2] showed that overexpression of a CIP4 variant (CIP4-V) can increase the tyrosine phosphorylation levels of β-catenin and cause the loss of cell–cell adhesion in renal cell carcinoma. In our investigation, E-cadherin loss was also observed in NRK-52E cells that overexpressed CIP4 that did not receive TGF-β1 stimulation, and CIP4 was shown to interact with β-catenin. In addition, in CIP4-overexpressed cells, increased expression of Snail1 was detected accompanied by the reduced expression of E-cadherin, the same result was also observed in TGF-β1 treated cells. Thus, it is possible that CIP4 may be critical for β-catenin-mediated cell–cell adhesion.

We postulated that CIP4 regulates E-cadherin expression by promoting translocation of β-catenin to the nucleus. β-Catenin is a mediator that regulates cadherin expression, as noted above. Yook* et al.* [25] showed that the Wnt signaling pathway may potentially stabilize β-catenin and increase *Snail* protein levels, which engage in the transcriptional processes of epithelial phenotype switching. β-Catenin also translocates to the nucleus and forms a complex with T cell factor (TCF); the complex then binds to the target gene *Slug* and represses the E-cadherin promoter [13]. As we knew that CIP4 interacted with β-catenin, we wondered if it had an effect on β-catenin nuclear translocation. We, therefore, then focused on nuclear proteins, and detected increased β-catenin expression in NRK-52E cells that overexpressed CIP4. The result was the opposite of that of the CIP4-siRNA knockdown group. Furthermore, β-catenin depletion was able to restore the expression of E-cadherin in cells transfected with CIP4 plasmid.

Our study thus provides evidence that nuclear translocation of β-catenin can regulate the expression of E-cadherin in epithelial cells, and that CIP4 binds with β-catenin and potentially mediates translocation of β-catenin to the nucleus where finally it regulates the expression of E-cadherin. From these findings, we propose in the future to identify the specific region of the CIP4-β-catenin interaction and the underlying mechanism by which CIP4 overexpression promotes nuclear translocation of β-catenin, and whether or not over-expressed CIP4 regulates the expression E-cadherin through Sail gene family. Further studies are also needed to evaluate CIP4 as a therapeutic target in alleviating the development of chronic kidney diseases. In addition, CIP4 is an interacting protein of Cdc42, a member of Rho-like GTPases [1]. The potential cross-talk between CIP4 and Rho-like GTPases and the Wnt and other intracellular signaling pathways warrants investigations in the future.

In summary, we found that CIP4 modulates the expression of E-cadherin in NRK-52E cells by regulating the nuclear translocation of β-catenin. Furthermore, E-cadherin expression was promoted by CIP4 overexpression and inhibited by CIP4 depletion. Our findings support the importance of CIP4 in regulating E-cadherin expression, and that CIP4 may be a potential therapeutic target in the treatment of chronic kidney diseases.

## 4. Materials and Methods

### 4.1. Ethics Statement

The use and care of animals employed in our renal fibrotic model were in accordance with guidelines for animal care at our university and all relevant laws of China. This study was approved by the ethics committee of Tongji Hospital, Tongji Medical College, Huazhong University of Science and Technology. Male adult Sprague-Dawley rats weighing 150 to 200 g were purchased from the Tongji Laboratory Animal Center (Wuhan, Hubei, China). Each rat was housed in our animal facility, received humane care under pathogen-free conditions, and was fed a standard laboratory diet with unlimited access to water.

### 4.2. Experimental Protocols

The rats were randomly divided into a 5/6 nephrectomized group (*n* = 12) and a sham-operated group (*n* = 12). Briefly, the animals were anesthetized with 2% pentobarbital sodium (50 mg/kg body weight) by intraperitoneal injection. Body temperature was monitored and maintained at 37 °C. A dorsoventral incision parallel to the spinal cord was made to expose the left kidney, and the upper and lower poles (two-thirds) of the left kidney were removed. Subsequently, the right kidney was exposed and removed one week later. For the sham operation, animals underwent the same surgical procedure as above without removing any renal mass. All rats were euthanized 20 weeks later, after nephrectomy. Kidneys were immediately excised and fixed with 4% paraformaldehyde. Renal tissues were stained with Masson’s trichrome for histological examination.

### 4.3. Cell Culture and Treatment

Cells of the normal rat renal tubular epithelial cell line NRK-52E (Cell Repository, Chinese Academy of Sciences, Shanghai, China) were cultured in Dulbecco’s modified Eagle’s medium/high glucose (Invitrogen; Carlsbad, CA, USA) supplemented with 10% fetal bovine serum (Gibco; Grand Island, NY, USA) and 1% penicillin/streptomycin. These cells were maintained in 5% CO_2_ at 37 °C. NRK-52E cells were cultured in serum-free medium for 12 h before being treated with 10 ng/mL TGF-β1 for 72 h (Peprotech; Rocky Hill, NJ, USA).

### 4.4. Western Blot Analysis

Cells were lysed in buffer (1% Triton X-100, 0.5% Nonidet P-40, 20 mM Tris-HCl, 15 mM NaCl, 1 mM EDTA, 1 mM egtazic acid, 1 mM Na_3_VO_4_·10H_2_O, 2 mM NaF, 2 mM Na_2_P_2_O_4_·10H_2_O, 10 mM β-glycerophosphate disodium salt (pH 8.0) and protease inhibitor cocktail (5 mM phenylmethylsulfonyl fluoride, 5 μg/mL leupeptin, 5 μg/mL pepstatin, and 5 μg/mL aprotinin). Nuclear proteins were obtained with a nuclear and cytoplasmic protein extraction kit (Beyotime Institute of Biotechnology; Shanghai, China). Protein concentration was determined by the Bradford protein assay. A 100-μg aliquot of protein was then subjected to SDS-polyacrylamide gel electrophoresis for separation and transferred onto polyvinylidene difluoride membranes. After transfer, the membranes were blocked with 5% non-fat milk and blotted. Primary antibodies were directed against CIP4 (Santa Cruz Biotechnology; Santa Cruz, CA, USA), β-catenin (Abcam; Cambridge, MA, USA), E-cadherin and Lamin A/C (BD Biosciences; San Jose, CA, USA), and GAPDH and β-actin (Proteintech Group; Chicago, IL, USA). The bound antibody complexes were visualized by enhanced chemiluminescence (SuperSignal West Femto Kit, Pierce; Rockford, IL, USA), and quantitated by volume densitometry using Quantity One software (Alpha Innotech; San Leandro, CA, USA) with normalization to one of GAPDH, β-actin (Actb), or Lamin A/C.

### 4.5. Establishment of Stable CIP4-Overexpressing NRK-52E Clones

To obtain overexpression of CIP4, NRK-52E cells were transfected with CIP4-pcDNA4.0 using Lipofectamine 2000 (Invitrogen) according to the manufacturer’s instructions. Cells were plated at 90% confluence in 6-well plates without antibiotics one day before transfection. These cells were washed and seeded in growth medium with Zeocin for 14 days to enable selection. Zeocin-resistant clones were then harvested and analyzed.

### 4.6. RNA Interference

To silence the expression of CIP4 and β-catenin, short-interfering RNA (siRNA) specific for CIP4 and β-catenin of rats, and control siRNA, were purchased from Ribobio (Guangzhou, China). NRK-52E cells were transfected with siRNA by using Lipofectamine 2000 (Invitrogen) according to the manufacturer’s instructions. Cells were analyzed 72 h after transfection.

### 4.7. Immunoprecipitation

Cells were lysed as described above. Equal volumes of cell lysates were pre-incubated with anti-CIP4 antibodies (Santa Cruz Biotechnology; Santa Cruz, CA, USA) at 4 °C for 1 h and then incubated overnight with 20 μL Protein A/G beads (Santa Cruz Biotechnology). The beads were then washed 4× with lysis buffer, boiled in Laemmli loading buffer, and protein was collected and immunoblotted with anti-β-catenin and anti-CIP4 antibodies as described above.

### 4.8. Immunohistochemistry

For immunohistochemical studies, paraffin sections were incubated with primary anti-CIP4 antibody at 4 °C overnight. The sections were then incubated with biotinylated goat anti-rabbit IgG antibody as the secondary antibody, and antibody reactions were visualized by using diaminobenzidine (DAKO; Tokyo, Japan). Images from the microscope were captured with a Nikon DXM1200 digital camera.

### 4.9. Statistical Analyses

Results are expressed as mean ± s.d. Data were analyzed by *t*-tests and one-way analysis of variance (ANOVA) followed by a *post-hoct*-test. Analyses were performed using SPSS software (version 15.0). Significance was assessed at *p* < 0.05.

## References

[1] Aspenstrom P. (1997). A Cdc42 target protein with homology to the non-kinase domain of FER has a potential role in regulating the actin cytoskeleton. Curr. Biol..

[2] Tsuji E., Tsuji Y., Fujiwara T., Ogata S., Tsukamoto K., Saku K. (2006). Splicing variant of Cdc42 interacting protein-4 disrupts beta-catenin-mediated cell–cell adhesion: Expression and function in renal cell carcinoma. Biochem. Biophys. Res. Commun..

[3] Banerjee P.P., Pandey R., Zheng R., Suhoski M.M., Monaco-Shawver L., Orange J.S. (2007). Cdc42-interacting protein-4 functionally links actin and microtubule networks at the cytolytic NK cell immunological synapse. J. Exp. Med..

[4] Zhang Y.E. (2009). Non-Smad pathways in TGF-β signaling. Cell Res..

[5] Kardassis D., Murphy C., Fotsis T., Moustakas A., Stournaras C. (2009). Control of transforming growth factor β signal transduction by small GTPases. FEBS J..

[6] Miyazono K. (2009). Transforming growth factor-β signaling in epithelial-mesenchymal transition and progression of cancer. Proc. Jpn. Acad. Ser. B Phys. Biol. Sci..

[7] Tian X., Liu Z., Niu B., Zhang J., Tan T.K., Lee S.R., Zhao Y., Harris D.C., Zheng G. (2011). E-cadherin/β-catenin complex and the epithelial barrier. J. Biomed. Biotechnol..

[8] Meng W., Takeichi M. (2009). Adherens junction: Molecular architecture and regulation. Cold Spring Harb. Perspect. Biol..

[9] Baum B., Georgiou M. (2011). Dynamics of adherens junctions in epithelial establishment, maintenance, and remodeling. J. Cell Biol..

[10] Niessen C.M., Leckband D., Yap A.S. (2011). Tissue organization by cadherin adhesion molecules: Dynamic molecular and cellular mechanisms of morphogenetic regulation. Physiol. Rev..

[11] Santibanez J.F., Quintanilla M., Bernabeu C. (2011). TGF-β /TGF-β receptor system and its role in physiological and pathological conditions. Clin. Sci..

[12] MacDonald B.T., Tamai K., He X. (2009). Wnt/β-catenin signaling: Components, mechanisms, and diseases. Dev. Cell.

[13] Nelson W.J., Nusse R. (2004). Convergence of Wnt, β-catenin, and cadherin pathways. Science.

[14] Liu Y. (2010). New insights into epithelial-mesenchymal transition in kidney fibrosis. J. Am. Soc. Nephrol..

[15] Truesdell P., Ahn J., Chander H., Meens J., Watt K., Yang X., Craig A.W. (2014). CIP4 promotes lung adenocarcinoma metastasis and is associated with poor prognosis. Oncogene.

[16] McCrea P.D., Gu D. (2010). The catenin family at a glance. J. Cell Sci..

[17] Zeng R., Han M., Luo Y., Li C., Pei G., Liao W., Bai S., Ge S., Liu X., Xu G. (2011). Role of Sema4C in TGF-β1-induced mitogen-activated protein kinase activation and epithelial-mesenchymal transition in renal tubular epithelial cells. Nephrol. Dial. Transplant..

[18] Zeng R., Yao Y., Han M., Zhao X., Liu X.C., Wei J., Luo Y., Zhang J., Zhou J., Wang S. (2008). Biliverdin reductase mediates hypoxia-induced EMT via PI3-kinase and Akt. J. Am. Soc. Nephrol..

[19] Galichon P., Finianos S., Hertig A. (2013). EMT-MET in renal disease: Should we curb our enthusiasm. Cancer Lett..

[20] Wilson P.D. (2011). Apico-basal polarity in polycystic kidney disease epithelia. Biochim. Biophys. Acta.

[21] Hills C.E., Squires P.E. (2010). TGF-β1-induced epithelial-to-mesenchymal transition and therapeutic intervention in diabetic nephropathy. Am. J. Nephrol..

[22] Lilien J., Balsamo J. (2005). The regulation of cadherin-mediated adhesion by tyrosine phosphorylation/dephosphorylation of β-catenin. Curr. Opin. Cell Biol..

[23] Medici D., Hay E.D., Olsen B.R. (2008). Snail and Slug promote epithelial-mesenchymal transition through β-catenin-T-cell factor-4-dependent expression of transforming growth factor-beta3. Mol. Biol. Cell.

[24] Wang H., Zhang G., Zhang H., Zhang F., Zhou B., Ning F., Wang H.S., Cai S.H., Du J. (2014). Acquisition of epithelial-mesenchymal transition phenotype and cancer stem cell-like properties in cisplatin-resistant lung cancer cells through AKT/β-catenin/Snail signaling pathway. Eur. J. Pharmacol..

[25] Yook J.I., Li X.Y., Ota I., Fearon E.R., Weiss S.J. (2005). Wnt-dependent regulation of the E-cadherin repressor snail. J. Biol. Chem..

